# Influence of Process Parameters on the Efficiency of Pervaporation Pilot ECO-001 Plant for Raw Ethanol Dehydration

**DOI:** 10.3390/membranes14040090

**Published:** 2024-04-14

**Authors:** Anna Kujawska, Wojciech Kujawski, Wiesław Capała, Urszula Kiełkowska, Marek Plesnar, Joanna Kujawa

**Affiliations:** 1 Faculty of Chemistry, Nicolaus Copernicus University in Toruń, 7 Gagarina Street, 87-100 Toruń, Polandulak@umk.pl (U.K.);; 2 Łukasiewicz Industrial Chemistry Institute, 8 Rydygiera Street, 01-793 Warszawa, Poland; wieslaw.capala@ichp.lukasiewicz.gov.pl (W.C.);

**Keywords:** ethanol dehydration, pervaporation, poly(vinyl alcohol) composite PERVAP^TM^ 2200 membrane, pervaporation pilot plant ECO-001

## Abstract

Pervaporation is a membrane-based process used for the separation of liquid mixtures. As this membrane process is governed by the differences in the sorption and diffusivities of separated components, close boiling mixtures and azeotropic mixtures can effectively be separated. The dehydration of ethanol is the most common application of hydrophilic pervaporation. The pilot scale properties of hydrophilic composite poly(vinyl alcohol) PVA membrane (PERVAP^TM^ 2200) in contact with wet raw bioethanol are presented. The wet raw bioethanol was composed of ethanol (82.4–89.6 wt%), water (5.9–8.5 wt%), methanol (2.3–6.9 wt%), cyclohexane (0.2–2.4 wt%), higher alcohols (0.2–1.3 wt%), and acetaldehyde (0.004–0.030 wt%). All experiments were performed using a SULZER ECO-001 plant equipped with a 1.5 m^2^ membrane module. The efficiency of the dehydration process (i.e., membrane selectivity, permeate flux, degree of dehydration) was discussed as a function of the following parameters: the feed temperature, the feed composition, and the feed flow rate through the module. It was found that the low feed flow rate influenced the dehydration efficiency as the enthalpy of evaporation caused a high temperature drop in the module (around 25 °C at a feed flow rate equal to 5 kg h^−1^). The separation coefficient during pervaporation was in the range of 600–1200, depending on the feed composition. The increase in temperature augmented the permeation flux and shortened the time needed to reach the assumed level of dehydration. It was revealed that dehydration by pervaporation using ECO-001 pilot plant is an efficient process, allowing also to investigate the influence of various parameters on the process efficiency.

## 1. Introduction

Ethanol (EtOH) is an important and commonly applied solvent in the industry. The main markets for EtOH production are beverage manufacturing, production of pharmaceuticals, cosmetics, manufacturing of inks, detergents, and polymers [1,2]. However, owing to the COVID-19 pandemic, ethanol is widely applied as a disinfectant [3]. In addition, EtOH is a commonly used extractant of active compounds applied in various industries [4]. Ethanol is also an attractive compound applied as a fuel additive, owing to the fact that EtOH possesses several key properties, such as a motor octane number of 102 and an energy density of 19.6 MJ dm^3^ [5]. Moreover, the heat of combustion of EtOH equals 26,800 kJ kg^−1^ [6], which proves its high potential as a fuel. Ethanol can be also blended with gasoline and the addition of ethanol to gasoline improves its octane number [7]. Assuming the complete combustion of alcohol, one kilogram of EtOH can produce almost 3.0 × 10^7^ J, which makes it an effective fuel additive [8]. Nowadays, gasoline containing a 10% ethanol additive is commonly present in the U.S. and Brazilian petrol stations [9,10]. Since the beginning of 2024, it was also introduced in EU gasoline. Moreover, the application of fuel containing ethanol additive of 5–85% does not require any modification of existing engines [11]. It has to be underlined that the practical application of ethanol in the petroleum industry is possible if the fuel has been dehydrated prior to use. Allowed water content in ethanol fuel is 0.2 vol%, according to EN 15376 European standard [12] or 1.0 vol% owing to US regulations (ASTM D 4806) [13,14].

Distillation is a well described and commonly utilized process used for the separation of liquid mixtures. However, classical distillation generates high energy consumption, which is responsible for 95% of operational expenditures [15]. It has to be pointed out that the separation of the water-ethanol mixture and the production of pure EtOH solvent cannot be performed by simple distillation, owing to the creation of an azeotropic mixture (95.6 wt% EtOH and 4.4 wt% H_2_O) [15,16,17,18]. To overcome the mentioned problem, several methods were designed to break the azeotrope, e.g., extractive distillation, pressure swing adsorption, and membrane pervaporation [1,7,8,17,19,20,21,22,23,24,25,26,27,28,29].

Extractive distillation (ExD) is usually applied during the separation of non-pressure-sensitive azeotropes [19]. ExD provides separation of complex mixtures owing to the introduction at the top of the column of an additional solvent (entrainer). The added component affects the activity coefficients of the separated mixture components at the liquid phase, modifying the relative volatilities of the mixture constituents [21,30].

Zhang et al. [20] described the selection of the most appropriate entrainer for ExD of ethanol/n-hexane and ethanol/cyclohexane azeotropes separation. The selection was based on selectivity, relative volatility, and the influence of the given entrainer on the azeotropic system phase behavior. Butyl propionate and butyl butanoate were selected as the most appropriate solvents to separate ethanol-solvent mixtures among all entrainer solvents tested in this work [20].

Kiss and Szuszwalak [14] applied dividing-wall columns (DWC) during ethanol dehydration carried out using extractive distillation (ExD) and azeotropic distillation (AD). The authors claimed that DWC can successfully intensify the efficiency of the distillation process owing to lower investment and operational costs as well as reduced equipment requirements. The process efficiency assessment was performed using Aspen Plus software and sequential quadratic programming (SQP) methods. Ethylene glycol and n-pentane were utilized as mass separating agents (MSA) during the dehydration of a mixture containing 85 mol% of ethanol. The application of the proposed methods allowed the production of ethanol of 99.8 wt% purity [14]. The performed simulation showed energy savings of 10–20% compared with typical extractive and azeotropic distillation setups. However, taking into account investment costs, the application of the proposed concept is more profitable when a new EtOH dehydration plant is created, owing to the fact that investment costs related to the modernization of the already existing plant would exceed potential profits [14].

Ethanol dehydration can also be effectively performed by an adsorption process using molecular sieves as adsorbents [8,17]. Adsorption takes advantage of the enrichment of the adsorbent surface by sorbed substances at an interface between solid and liquid [31]. Desorption is performed to regenerate adsorbent without significant losses of its adsorptive properties. However, the desorption step requires high temperature and/or low pressure, which affects significantly overall process costs [14]. As was mentioned, molecular sieves are the most commonly applied sorbents during ethanol dehydration owing to high water adsorptive properties (high affinity to water molecules) and high process efficiency [8]. Zeolite molecular sieves (Zeolite NaA) are distinguished as the most appropriate materials to be applied during ethanol dehydration [17]. It is worth noticing that adsorption is currently the most common technology to dehydrate ethanol at an industrial scale in Poland.

Seo et al. [7] proposed an absorption process with molecular-sieving carbon (MSC) for the concentration of EtOH during small-scale bioethanol production. The best performance towards ethanol was obtained during experiments with MSC 5A (ethanol absorption capacity of 0.163 g g^−1^). Adsorption temperature and ethanol content in the broth were pointed out as the main factors determining the process efficiency; whereas, during a desorption step, the final ethanol concentration and its recovery rate were significantly influenced by water recovery temperature. The proposed system enables the production of ethanol of concentration up to 98.5 wt% [7].

Simo et al. [32] investigated the kinetics of ethanol and water adsorption/desorption on 3A zeolite during the pressure swing adsorption process. Performed experiments revealed that water adsorption on the 3A zeolite was significantly higher compared to ethanol, proving that the mentioned adsorbent favorably adsorbs water. The determined selectivity parameter of the adsorbent used in this study was equal to ca. 900. At the same time, very low ethanol uptake was observed (ca. 30 mmol kg^−1^) [32].

The most advanced method for solvent dehydration is the pervaporation (PV) process [33,34,35,36,37,38,39,40,41,42,43,44,45,46]. Pervaporation allows for the separation of liquid mixtures that are difficult to separate by applying simple distillation, e.g., azeotropes [33]. PV is a membrane-based separation method utilizing the difference in chemical potential between feed and permeate as a process driving force [34,35,36]. The driving force can be generated by applying a vacuum (VPV), sweeping gas (SGPV), or lower temperature (thermopervaporation, TPV) on the permeate side [37,38,39,40,41,42,43]. Generally, membrane-based separation methods are recognized as low-energy-demanding processes [44].

Polymeric membranes are the most commonly utilized materials for pervaporation applications [37]. Castro-Munoz et al. [1] applied cross-linked poly(vinyl alcohol) (PVA) membranes incorporated with graphene oxide (GO) for ethanol dehydration (10/90 water/EtOH) with pervaporation. The authors claimed that the membrane containing 1 wt% of GO exhibited the best performance, reaching a separation factor of 263 and a total permeate flux of 0.137 kg m^−2^ h^−1^ at 40 °C feed temperature, which was a 75 % improvement in PV process efficiency obtained during experiments performed with pristine PVA membranes. The authors also performed an analysis of feed temperature on overall process efficiency, showing that higher transport through the membrane can be obtained at the increased temperature [1].

Wang and Tsuru [47] applied cobalt-doped silica (Co-SiO_2_) membranes during pervaporative EtOH dehydration. The pervaporation process performance depended on the change in the membranes’ treating temperature. Pervaporation experiments performed in contact with a 94 wt% aqueous ethanol solution revealed that an increase in annealing temperature from 350 to 550 °C caused a drop in total flux (J_t_) from 1.2 to 0.75 kg m^−2^ h^−1^. At the same time, the separation factor (β) value was improved from 65 to 1670. Taking into consideration the Pervaporation Separation Index (PSI) parameter, values of PSI changed from 77 kg m^−2^ h^−1^ to 1252 kg m^−2^ h^−1^, which proves higher PV performance during the application of the membrane treated at higher temperatures.

Cai et al. [29] applied membranes based on poly(vinyl alcohol) modified by Ti_3_C_2_T_x_ molecules during the pervaporative dehydration of ethanol. The best membrane selected among a series of the tested selective layers in this study possessed 3 wt% loading of the modifier molecules. The authors claimed that the addition of the modifier improves ethanol dehydration efficiency due to the fact that Ti_3_C_2_T_x_ promotes membrane crosslinking density, which results in higher process separation efficiency. However, increased membrane crosslinking density also resulted in lower permeate flux, which significantly reduced transport performance across the membranes. The process performance obtained during the application of the mentioned membrane was the following: total flux of 0.074 kg m^−2^ and separation factor of 2585 during pervaporation performed at 37 °C and at 93 wt% of ethanol content in the feed. The mentioned above J_t_ and β values corresponded to a PSI equal to 191 kg m^−2^ h^−1^.

**Figure 1:**
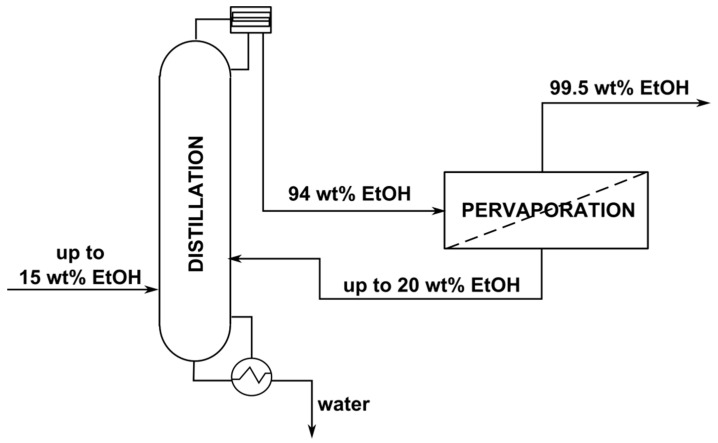
Scheme of the hybrid distillation-pervaporation system for ethanol dehydration (adapted with permission from [24]. Copyright © 2013, Elsevier).

To improve ethanol dehydration performance and overcome the drawbacks of distillation and pervaporation, researchers proposed the hybrid distillation pervaporation process (Figure 1).

Novita et al. [48] tested the performance of the extractive distillation (ExD) column coupled with pervaporation (PV) during the ethanol dehydration process. ExD glycerol was used as an entrainer. The PV operation was performed with a cellophane membrane and was aimed at separating the glycerol-water mixture. The performed simulation revealed that owing to the application of the hybrid ExD-PV configuration, it is possible to save up to 25% and 41% of total annual costs and energy expenditures, respectively, compared with the standard configuration (ExD combined with recovery column).

Kang et al. [49] investigated and compared the efficiency of 95 wt% ethanol dewatering using molecular sieves and pervaporation process with hydrophilic membranes (Dow Filmtech membrane—BW30XLE). PV flux and the concentration of water in permeate, obtained at 65 °C, were equal to 8.39 g m^−2^ s^−1^ and 53.3 wt%, respectively. The authors claimed that during pervaporation performed at 60 °C using a membrane unit containing 120 m^2^ membrane area, the same efficiency can be obtained as during ethanol dehydration by 160 tons of molecular sieve unit.

Leon et al. [50] proposed a distillation-pervaporation hybrid in a single unit (DPSU) that takes advantage of a pervaporation unit situated inside a distillation column. The DPSU system was applied during the separation of the ethanol-isopropanol-water mixture. In this study, the inverted separation order of isopropanol and water using the DPSU system was obtained, which was in contrast to the traditional distillation. Moreover, the removal of water by the pervaporation part of the system resulted in a reduction in the volatility of the lightest components of the mixture and shifted characteristic distillation points [50].

Ethanol from the fermentation broth contains typically 8 wt% to 12 wt% EtOH. The fermentation broth typically undergoes distillation allowing for the purification (removal of minor components like aldehydes or higher alcohols) and rectification leading to the near-azeotropic mixture. However, this mixture still contains around 5% water. Anhydrous ethanol for chemical and fuel uses is obtained either by entrainer distillation with cyclohexane or by adsorption on molecular sieves. Entrainer distillation is a relatively expensive method, and in addition, there is some concern on environmental and health grounds over the use of dehydrating agents. Hydrophilic pervaporation can also be considered as an appropriate and competitive replacement of entrainer distillation and adsorption on molecular sieves techniques.

This work aims to present the flexibility of both pervaporation itself and the flexibility of the PV ECO-001 (Sulzer/DeltaMem AG, Allschwil, Switzerland) setup used for the raw ethanol dehydration of various compositions. During experiments, different parameters influencing the dehydration process, like feed temperature, composition, and feed flow rate, were evaluated. The raw bioethanol taken from one of the distilleries located in the south of Poland was utilized as a feed mixture.

## 2. Experimental

The compositions of feed mixtures used in experiments are gathered in Table 1. Mixtures used in Run I and Run II contained higher amounts of methanol, alcohol (C3–C5), and cyclohexane. The ethanol content in feed mixtures used in Run III and Run IV was around 89 wt%. The water content was in the range of 5.9 (Run II) to 8.5 wt% (Run IV)

Permeate flux was determined by weight, whereas the product and permeate compositions were determined by using a gas chromatograph GC (VARIAN 3300), Varian Analytical Instruments Inc. (Walnut Creek, CA, USA). The GC was equipped with the Porapak Q, packed column (Agilent Technologies Inc., Santa Clara, CA, USA) using helium as carrier gas and a TCD—a thermal conductivity detector. The TCD allows for direct evaluation of water content in the analyzed liquid mixture. The conditions for GC measurements were the following: injection port temperature was set at 200 °C, the detector temperature at 220 °C, and the column temperature at 180 °C. BORWIN v. 1.21.05 software (JMBS, Grenoble, France) was used for data acquisition and processing. The accuracy of the experiments was in the range of ±5%.

To evaluate the pervaporation performance, total flux (J_t_) and separation factor (β) were employed using Equations (1) and (2).

(1) $$J_{t}=\frac{W}{A\times t}$$

(2) $$\beta =\frac{Y_{e}/Y_{w}}{X_{e}/X_{w}}$$

where W is the permeate weight (g), A is the effective area of the membrane (m^2^), and t is the experiment duration (h). Y_e_ and Y_w_ are weight fractions of ethanol (e) and water (w) in permeate, respectively; whereas, X_e_ and X_w_ are weight fractions of ethanol (e) and water (w) in feed, accordingly.

Partial fluxes (J_i_) were calculated using Equation (3)

(3) $$J_{i}=J_{t}\times Y_{i}$$

where J_t_ is total flux and Y_i_ is a weight fraction of component i in permeate.

**Figure 2:**
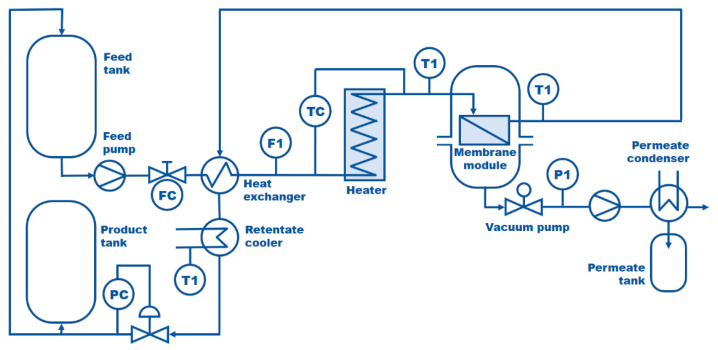
Scheme of the Pervaporation ECO-001 plant.

Pervaporation experiments were carried out in the ECO-001 Pervaporation Unit (Sulzer/DeltaMem AG, Allschwil Switzerland). The scheme of this unit is presented in Figure 2. The ECO-001 plant is equipped with a plate-and-frame membrane module containing 1.5 m^2^ of PERVAP^TM^ 2200 membrane. According to the literature and producer data, PERVAP^TM^ 2200 can be utilized for the removal of water from the mixtures of organic solvents as well as for the dewatering of azeotropic mixtures containing up to 15 wt% of water [45]. PERVAPTM 2200 is a hydrophilic PVA-based composite membrane. In general, Pervap^TM^ hydrophilic membranes are multilayer structures, consisting of at least three different layers [45]:

i. A thin selective skin layer is prepared using cross-linked PVA. This top layer is responsible for the membrane selectivity and permeability. The mode of PVA deposition and crosslinking degree determines the membrane properties. The thickness of this layer is up to 10 μm.
ii. Intermediate ultrafiltration support. This layer (40–60 μm thick) is prepared by a phase inversion method usually from polyacrylonitrile (PAN).
iii. The polyester non-woven backing fabric. This layer (80–120 μm thick) does not influence either selectivity or permeate flux.

ECO-1 unit was specially designed for pervaporation tests exceeding the laboratory scale or for the treatment of small volumes up to 120 L/day, depending on solvent, amount of water to be removed and final water content to be reached. The batch-wise mode of the operation was chosen to perform the dehydration experiments. The thermostated feed solution circulated in the system for a given period of time needed to reach the assumed level of dehydration. The permeate was cooled down and collected in the permeate tank whereas the retentate was returned to the feed tank (Figure 2).

The dehydration experiments were performed for 4 different mixtures taken directly from the industrial plant producing dehydrated ethanol by entrainer distillation. Compositions of the feed mixtures used in experiments are summarized in Table 1, whereas other process parameters are presented in Table 2.

## 3. Results and Discussion

**Figure 3:**
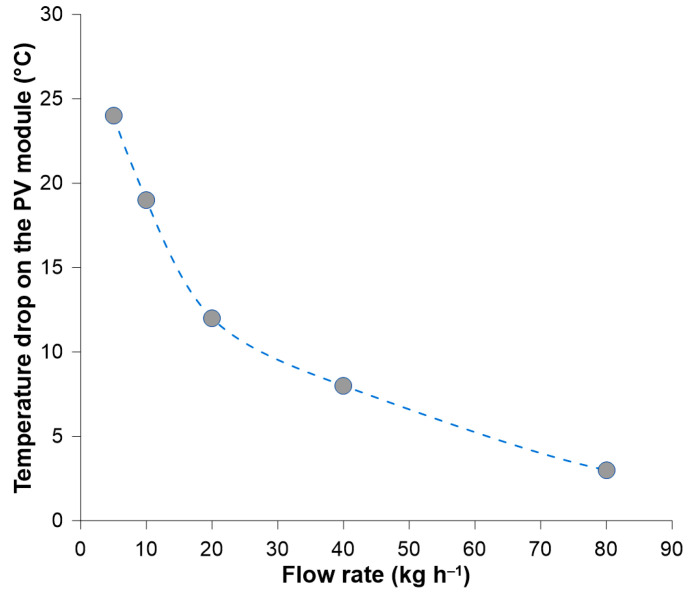
Run I—the temperature drop on the pervaporation module as a function of the feed flow rate.

Run I was performed to get information on the relation between the feed flow rate and the temperature drop on the pervaporation module. It was found that by applying low circulation rates, the temperature drop on the module was around 10–25 ° C (Figure 3). This temperature drop was caused both by the enthalpy of permeate evaporation and the heat dissipation to the environment. The decrease of the temperature drop can be achieved by increasing the feed circulation rate through the module. Therefore, to keep the temperature drop as small as possible, the feed circulation rate of 80 kg h^−1^ was chosen for Runs II, III, and IV.

**Figure 4:**
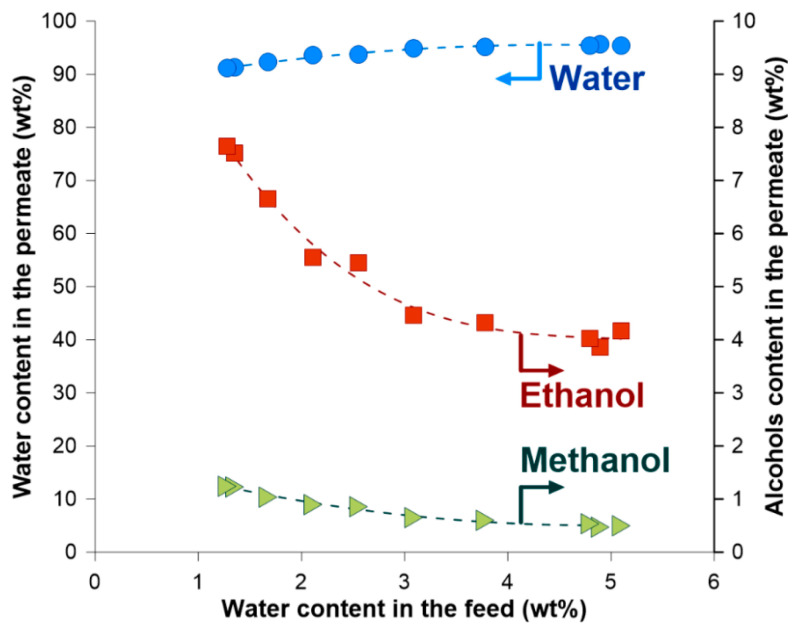
Composition of the permeate mixture as a function of water content in the feed/retentate mixture (Run II). Note, that with the progress of the dehydration process water content in the feed/retentate is decreasing.

The separation characteristics of the PERVAP^TM^ 2200 membrane used in the ECO-001 plant is presented in Figure 4. It is seen that water was preferentially transported through the membrane. With a decreasing amount of water in the feed mixture, the amount of methanol and ethanol in the permeate increases. It is worth underlining that neither higher alcohols nor cyclohexane were found in the permeate.

Runs II and III were performed for the feed mixtures with different compositions (Table 1). The feed mixture in Run III contained more water and more ethanol than the feed mixture used in Run II. The temperature of the feed mixture in Run II was 77 °C; whereas it was equal to 82 °C in Run III (Table 2).

**Figure 5:**
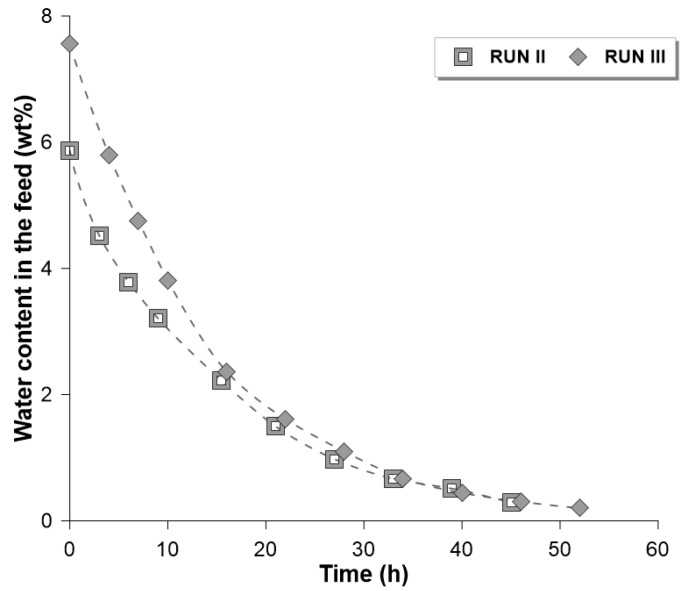
Water content in the feed/retentate mixture as a function of the dehydration time.

Figure 5 presents the efficiency of the PERVAP^TM^ 2200 membrane in ethanol dehydration during Runs II and III. The final water content in Run II was 0.3 wt% with the degree of water removal equal to 95%, whereas the final water content in Run III was as low as 0.2 wt% with the degree of water removal equal to 97.4%.

**Figure 6:**
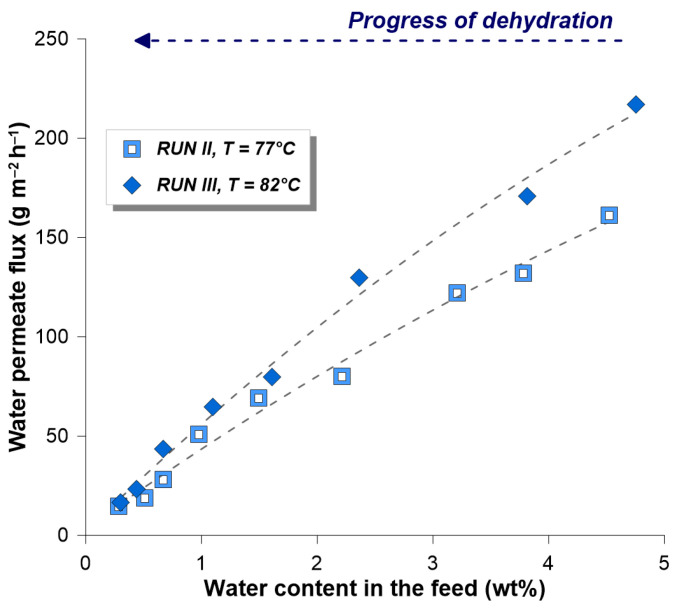
Water permeate flux as a function of water content in the feed/retentate mixture. Note that with the progress of the dehydration process water content in the feed/retentate is decreasing.

Comparing the influence of the process temperature on the dehydration efficiency, it can be stated that for the same range of dehydration (i.e., from 5.8 wt% down to 0.3 wt%) the time needed to perform such process was shorter in Run III. The higher efficiency of the dehydration at higher temperatures is caused mainly by the increase of the permeate flux. Figure 6 compares the water permeate fluxes during Runs II and III. It is worth noting that with the temperature higher of 5 °C in Run III, the permeate fluxes were ca. 30% higher than those in Run II. For instance, in contact with a feed mixture containing 3 wt% water, the permeate flux in Run II was equal to 113 g m^−2^ h^−1^, compared to 148 g m^−2^ h^−1^ in Run III.

Experiments performed in Runs I, II, and III allowed for the choice of the optimum process parameters when using the ECO-001 pervaporation unit for the dehydration process. Run IV was performed at a temperature of 90 °C and the feed circulation rate was equal to 80 kg/h. The feed mixture contained initially 8.5 wt% of water (Table 1 and Table 2).

**Figure 7:**
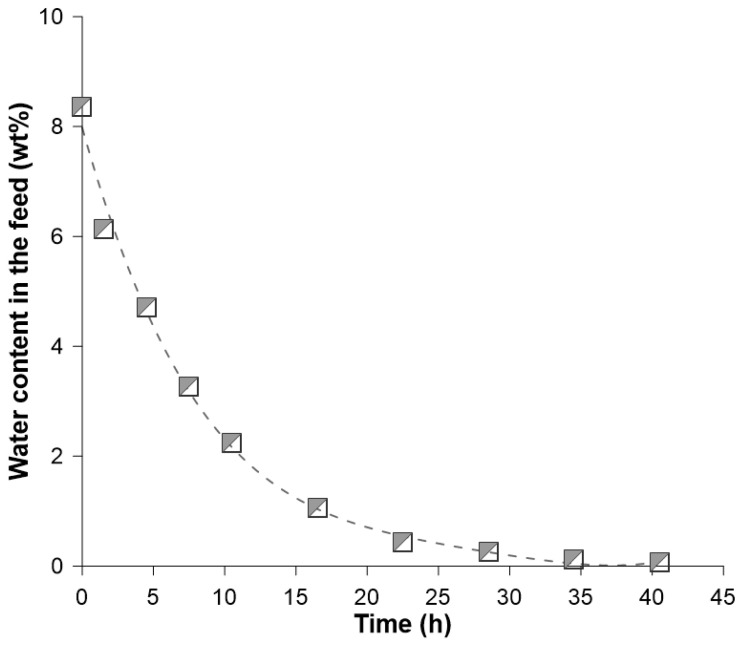
Water content in the feed/retentate mixture as a function of the pervaporation duration (Run IV).

The efficiency of the dehydration process is presented in Figure 7. The decrease of water content from 8.5 to 1.0 wt% needed 17 h of the batch process, whereas from 1.0 wt% to 0.1 wt% needed an additional 23 h (Figure 7). It suggests that the deep dehydration of ethanol would need more time or a larger membrane area. The degree of water removal in Run IV reached 99% at the end of the process (40 h of the batch process).

**Figure 8:**
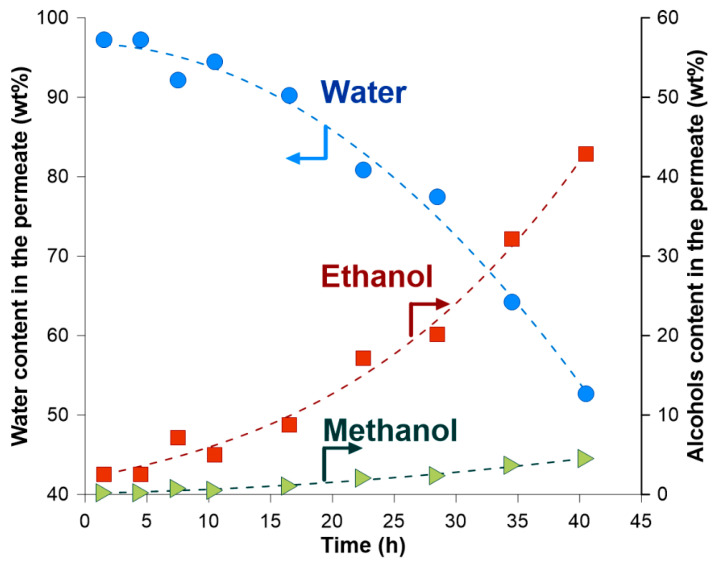
Permeate composition as a function of the dehydration time (RUN IV).

The separation properties of the PERVAP^TM^ 2200 membrane during Run IV are presented in Figure 8, and it was expressed by presenting the composition of the permeate vs. time of dehydration process. It is seen that the amount of water in the permeate decreased over the time of the dehydration process, whereas the total alcohol content in the permeate increased. When the membrane contacted feed containing 0.1 wt% of water, the permeate contained 43 wt% of EtOH and 4.5 wt% of MeOH; however, the amount of the permeate was very small. It should be stressed that the average composition of the permeate collected during 40 h of the dehydration process was the following: 93.6 wt% water, 5.8 wt% of EtOH, and 0.6 wt% of MeOH. Moreover, high values of the separation factor β were found for all performed runs. The average separation factor for the feed water content in the range 1–6 wt% was around 700.

**Figure 9:**
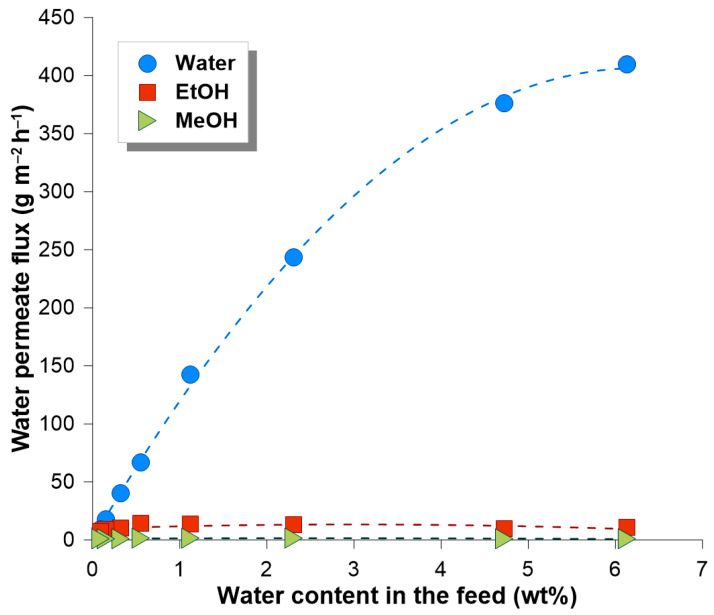
Permeate fluxes as the function of water content in the feed/retentate mixture (Run IV). Note, that with the progress of the dehydration process water content in the feed/retentate is decreasing.

The transport properties of the PERVAP^TM^ 2200 membrane during Run IV are presented in Figure 9. It is seen that the water permeate flux decreases with decreasing the amount of water in the feed mixture. The average permeate flux of water was equal to 280 g m^−2^ h^−1^. The flux of ethanol and methanol through PERVAP^TM^ 2200 was in the range of 15 g m^−2^ h^−1^ (EtOH) and 2 g m^−2^ h^−1^ (MeOH). For the water content in the feed mixture below 1 wt% both ethanol and methanol fluxes also decreased.

## 4. Conclusions

The results obtained during this research allowed for the following conclusions:

- During the dehydration of ethanol by using the pervaporation ECO-001 unit, up to 99 wt% of water was removed from the feed mixture, reaching the final water content in the product solution equal to 0.1 wt% (average separation factor β was equal to 700). It has to be underlined that pervaporation allows one to obtain an even lower level of water content in the feed mixture; however, in such a case, the PV process should be performed longer. Moreover, an additional increase in the process efficiency would be obtained if the modules equipped with membranes of larger membrane areas were applied.
- The feed temperature and the initial water content were the most important parameters influencing the efficiency of the process. The permeate flux increased substantially with temperature increase, reducing the time necessary to reach the assumed final water level.
- Ethanol and methanol as polar components permeated also through the membrane; however, the cumulative content of both alcohols in the permeate was lower than 10 wt%.
- The ECO-001 unit equipped with hydrophilic PERVAP^TM^ membranes is an ideal approach to test various organic solvents for dehydration applications. Nowadays, pervaporation can be used as the simple and efficient method for ethanol dehydration either as the separated method or coupled with an existing dehydration system (i.e., entrainer distillation or molecular sieves units).

## Figures and Tables

**Table 1:** Composition of feed mixtures used in the experiments.

	Feed Composition [wt%]			
Component	Run I	Run II	Run III	Run IV
Water	6.97	5.87	7.56	8.46
Methanol	6.81	6.86	2.40	2.29
Ethanol	82.45	84.63	89.64	88.83
Higher alcohols (C3–C5)	1.35	0.86	0.224	0.184
Acetaldehyde	0.020	0.027	0.005	0.004
Cyclohexane	2.40	1.76	0.17	0.23

**Table 2:** Process parameters used in the evaluation of the efficiency of the pervaporation dehydration process utilizing the PERVAP^TM^ 2200 PVA-based membrane.

Run ID	Amount of the Feed [kg]	Feed Circulation Rate [kg/h]	Feed Temperature [°C]
I	140	5–80	70–77
II	77	80	77
III	82	80	82
IV	87	80	92

## Data Availability

The original contributions presented in the study are included in the article; further inquiries can be directed to the corresponding author.

## Nomenclature

AD	Azeotropic distillation
Co-SiO_2_	Cobalt doped silica
DPSU	Distillation-Pervaporation in a Single Unit
DWC	Dividing-wall columns
EtOH	Ethanol
E-DWC	Extractive dividing-wall column
ExD	Extractive distillation
GO	Graphene oxide
MSA	Mass separating agents
MSC	Molecular-sieving carbon
PSI	Pervaporation Separation Index
PV	Pervaporation
PVA	Poly (vinyl alcohol)
SGPV	Sweeping gas pervaporation
SQP	Sequential quadratic programming
TPV	Thermopervaporation
VPV	Vacuum pervaporation
